# Prevalence, Molecular Characterisation and Antimicrobial Resistance of *Enterococcus faecalis* and *Enterococcus faecium* Isolated from Bovine Mastitis in Poland

**DOI:** 10.3390/pathogens15060613

**Published:** 2026-06-08

**Authors:** Ewa Zastempowska, Magdalena Twarużek, Jan Grajewski, Henryka Lassa

**Affiliations:** 1 Department of Physiology and Toxicology, Faculty of Biological Sciences, Kazimierz Wielki University, 85-064 Bydgoszcz, Poland; twarmag@ukw.edu.pl (M.T.); jangra@ukw.edu.pl (J.G.); 2 Milk Testing Laboratory, 85-065 Bydgoszcz, Poland; lassa.henryka@gmail.com

**Keywords:** *Enterococcus*, mastitis, virulence genes, antimicrobial resistance, vancomycin resistance, macrolide resistance, tetracycline resistance

## Abstract

Enterococci are among the most frequently isolated environmental bacteria that cause mastitis in cows. This study aimed to determine the prevalence of virulence genes, as well as phenotypic and genotypic antibiotic resistance, among eighty enterococcal isolates from cases of bovine mastitis in Polish herds. The presence of virulence and antibiotic resistance genes was determined by PCR. *E. faecalis* isolates were found to carry more virulence genes than *E. faecium* isolates, including the *efaA*_fs_ (100%), *ace* (98.1%), *gelE* (86.5%), *asa1* (63.5%), *esp* (57.7%) and *cylA* (17.3%) genes. The *efaA*_fm_ gene was the only virulence gene detected in *E. faecium* isolates. This study revealed that *E. faecalis* showed a higher virulence gene burden. The *erm*B gene was present in 90.9% of the *Enterococcus* spp. that were phenotypically resistant to erythromycin. Almost all tetracycline-resistant *Enterococcus* isolates carried the *tet*(M) gene (94.3%), either alone or in combination with the *tet*(L) and *tet*(O) genes. Three isolates harboured *van*C genes and were susceptible to vancomycin (MIC = 4 μg/mL). The results confirm the high level of antimicrobial resistance of enterococci isolated from cows with mastitis and indicate the genes that may be responsible for this resistance.

## 1. Introduction

Enterococci are pathogens that contribute to bovine mastitis. It is estimated that they cause approximately 1.5–21% of clinical and subclinical mastitis [1,2,3,4,5]. However, it is possible that some enterococci are misidentified based solely on phenotypic characteristics [6]. Misidentification of the causative agent of mastitis may lead to inappropriate clinical action [7]. *Enterococcus* spp. and esculin-hydrolysing streptococci isolated from the bovine mammary gland are often treated as a homogeneous group of microorganisms. However, enterococci demonstrate higher minimum inhibitory concentration (MIC) values for some antibiotics, e.g., to ampicillin and penicillin due to intrinsic resistance [8,9]. This highlights the need to identify microorganisms isolated from milk using more accurate methods, such as DNA-based molecular (e.g., 16S rRNA sequencing, PCR, whole genome sequencing; WGS) and proteomic methods (e.g., matrix-assisted laser desorption/ionization time-of-flight mass spectrometry; MALDI-TOF) [7,10,11].

*E. faecalis* and *E. faecium* are the enterococcal species most commonly isolated from cows with mastitis [3,4,9,12,13,14]. According to Current Concepts of Bovine Mastitis, both species are important mammary gland pathogens [11]. Enterococci are characterised by the presence of potential virulence factors that favour colonisation, such as an aggregation substance (AS), a collagen-binding protein (Ace), an endocarditis antigen (EfaA) and a surface protein (Esp), and affect host tissues, such as a cytolysin (Cyl), a gelatinase (GelE) and a hyaluronidase (Hyl) [15,16]. Furthermore, *Enterococcus* spp. can form biofilms [17,18].

Antibiotic therapy is a common method of treating and controlling bovine mastitis; therefore, mastitis is the main reason for the use of antibiotics on dairy farms [19,20,21]. However, there has recently been a greater emphasis on the prudent use of antibiotics when treating food-producing animals, including those with mastitis [21,22]. Inappropriate antibiotic selection when treating bovine mastitis can contribute to the emergence and spread of antibiotic-resistant strains, hence the need for the careful use of antibacterial agents [23]. In vitro antibiotic susceptibility testing of the causative agent of mastitis facilitates the selection of appropriate therapy and enables continuous monitoring of the potential antibiotic resistance of pathogens [24,25]. Treatment of bovine mastitis is often initiated before the in vitro antibiotic susceptibility of the isolate is determined. In such cases, the choice of therapy is based on the available information on the general susceptibility of mastitis pathogens [26]. It is worth emphasising that the sensitivity of mastitis pathogens to antibiotics can vary by country and region [27]. This is due to legal regulations regarding the use of veterinary drugs in different countries and the availability of antibiotics [28].

Contaminated and unpasteurised milk from animals with mastitis, particularly subclinical cases, can be a source of microorganisms, including enterococci. When these enter the human food chain, they can pose a threat to human health due to their potential virulence factors and antibiotic resistance determinants [29,30]. Additionally, the growing consumer demand for natural, unprocessed foods, such as artisan cheeses produced from unpasteurised milk, poses a risk of infection with foodborne pathogens. In addition to the risks associated with consuming raw milk, enterococci from bovine mastitis can pose an occupational hazard to farmers and veterinarians. There is also a potential risk to individuals in close contact with farm animals, particularly those with weakened immune systems [31]. Enterococcus bacteria demonstrate a high rate of intrinsic tolerance/low-level resistance to many classes of antibiotics and can acquire multi-resistance mechanisms [32]. This limits the number of antibiotics available for treating human infections. Another problem is the ease with which virulence and resistance genes can be transmitted to other pathogenic bacteria. These properties make enterococcal infections a serious public health problem [20].

Although some authors have reported on the prevalence of virulence and resistance genes in enterococcal isolates from countries such as the USA, Brazil, South Korea, China and Romania, information on the genotypic characterisation of enterococci as causative agents of bovine mastitis in Europe, including Poland, is limited. Previously published Polish studies on enterococci from bovine milk focused only on the phenotypic resistance of isolates to antibiotics. The assessment of virulence and antimicrobial resistance gene profiles of bovine *Enterococcus* spp. isolates could fill a knowledge gap on the potential pathogenicity of these pathogens for cows. The aim of this study was therefore to investigate the virulence genes and antibiotic resistance determinants of *Enterococcus* spp. isolates that caused bovine mastitis in Poland.

## 2. Materials and Methods

### 2.1. Bacterial Isolates and Sample Collection

A total of 80 *Enterococcus* isolates (52 *E. faecalis*, 11 *E. faecium*, one *E. gallinarum*, two *E. casseliflavus* and 14 isolates of other species) were obtained from the inflammatory secretions of the bovine mammary glands of cows diagnosed with clinical and subclinical mastitis during the previously described study [5]. The samples were collected at different times between 2009 and 2017. The bacteria were isolated from 80 cows kept on 78 farms located in different regions of Poland. To avoid duplication of isolates, only one isolate belonging to a particular species from the same farm was used in the study. Mastitis diagnosis, milk sample collection and milk microbiological examination were performed as previously described [5]. Further phenotypic identification of enterococci was carried out using a serological test (Phadebact Strep D 100 Test; Bactus AB, Huddinge, Sweden) and API biochemical tests (bioMérieux, Marcy-l’Étoile, France). Additionally, enterococcal growth was assessed on a selective medium (Kanamycin esculin azide agar; Merck KGaA, Darmstadt, Germany), which is intended for the isolation of enterococci and group D streptococci, after 24 h incubation at 37 °C in aerobic conditions. Although 16S rDNA sequencing and the MALDI-TOF MS technique are currently considered the standard for bacterial identification [10], these methods are still too expensive for many diagnostic laboratories. Despite excellent sensitivity, rapidity, accuracy and low reagent costs of MALDI-TOF MS technique, the device itself is relatively expensive [33].

### 2.2. Reference Strains

In addition to the field enterococcal isolates, reference strains (LGC Standards, London, UK) were used in the study (Table 1). Both the tested isolates and the reference strains were stored in cryovials (Microbank; Pro-Lab Diagnostics, Richmond Hill, ON, Canada) at −70 °C.

### 2.3. DNA Isolation and Gene Detection Using PCR

The DNA of reference strains and enterococcal field isolates was extracted as previously described [34]. The isolates were identified to genus and species level using conventional PCRs (Table 2), after which all enterococci were tested for genes encoding virulence (Table 3) and antibiotic resistance (Table 4) factors. The reaction mixtures, chemicals and thermocycler were the same as those used previously [34], with modifications to the MgCl_2_ concentration and primer concentration (0.25 µM for *vanB*). The PCR conditions were optimised based on the original references. Primers for the *efaA*_fs_ and *efaA*_fm_ genes were designed based on the GenBank database under accession numbers: U03756 and AF042288, respectively. The primer sequences, PCR conditions and MgCl_2_ concentrations used in the study are included in Table 2, Table 3 and Table 4. A positive control (1 µL of DNA from a gene-positive strain) and a negative control (1 µL of nuclease-free water) were included in all PCR runs. Electrophoresis and DNA visualisation were carried out as previously described [34].

### 2.4. Antimicrobial Susceptibility Testing

The antimicrobial susceptibility of enterococci was tested using the disk diffusion method in Mueller–Hinton II Agar (Graso Biotech, Jabłowo, Poland), in accordance with the guidelines of the Clinical and Laboratory Standards Institute [43]. The following antimicrobial susceptibility discs were used: amoxicillin (AML; 25 µg), ampicillin (AMP; 10 µg), bacitracin (B; 10 u), cephalexin (CL; 30 µg), cefoperazone (CFP; 30 µg), erythromycin (E; 15 µg), cloxacillin (OB; 5 µg), lincomycin (MY; 15 µg), neomycin (N; 30 µg), penicillin G (P; 10 u), tetracycline (TE; 30 µg) (Oxoid Ltd. Basingstoke, Hampshire UK) and cefapirin (CPR; 30 µg) (Mast Diagnostics, Mast Group Ltd., Merseyside, UK). The test results were classified as sensitive (S), intermediate (I) or resistant (R) based on CLSI criteria [43] and information provided by the antibiotic manufacturers.

For enterococcal isolates with vancomycin resistance genes (*vanC1*, *vanC2/3*), the minimum inhibitory concentration (MIC) of vancomycin was determined using the broth microdilution method in accordance with the CLSI guidelines [43]. The results were interpreted based on CLSI [43] cut-off values, as well as data from the literature [38,44].

### 2.5. Statistical Analysis

Statistical analysis was performed with Microsoft Excel 2019 (Microsoft Corporation, Redmond, Washington, DC, USA) and Statistica version 13.3 (Microsoft 2020, Statsoft 2024). To determine whether there are statistically significant differences in the presence of specific virulence genes between clinical and subclinical mastitis, a chi-squared test using a contingency coefficient was performed at a significance level of alpha = 0.05. However, given the small number of subclinical cases (n = 7), the test may have low statistical power when interpreting non-significant associations.

## 3. Results

### 3.1. Type of Haemolysis on a Blood Medium

All *E. faecium* isolates exhibited α-haemolysis on an agar medium supplemented with 5% sheep blood, whereas most *E. faecalis* isolates exhibited γ-haemolysis (86.5%). Among the remaining *Enterococcus* bacteria, including *E. gallinarum* and *E. casseliflavus*, both α- and γ-haemolysis were observed. No β-haemolysis was demonstrated in any of the *Enterococcus* isolates tested (Table 5).

### 3.2. Occurrence of Virulence Genes

The study revealed that *E. faecalis* isolates carried the *ace*, *asa1*, *cylA*, *efaA*_fs_, *esp* and *gelE* genes. The *efaA*_fs_ gene was present in all isolates of *E. faecalis* tested, while the *ace*, *asa1*, *cylA*, *esp* and *gelE* genes occurred at varying frequencies. There was no statistically significant association between the presence of the tested virulence genes and the type of mastitis (Table 6).

Nine virulence gene profiles were identified in *E. faecalis* isolates. The most common profile was the *ace asa1 efaA*_fs_ *esp gelE* genotype, which was present in 21.2% of isolates (Table 7).

All examined *E. faecium* isolates contained the *efaA*_fm_ gene. Enterococci belonging to species other than *E. faecalis* and *E. faecium* did not harbour any of the tested virulence genes.

Using primers designed to amplify a fragment specific to the *E. faecalis* EfaA protein gene (*efaA*_fs_), amplification products of the expected size (569 bp) were obtained only in *E. faecalis* isolates and the *E. faecalis* ATCC 29212 reference strain. In contrast, a second pair of primers designed to amplify a fragment specific to the *E. faecium* EfaA protein gene (*efaA*_fm_) enabled a 361 bp amplicon to be obtained only in *E. faecium* isolates and the *E. faecium* ATCC 51559 reference strain. No PCR products were obtained when the above primer pairs were used with samples containing DNA from other *Enterococcus* species.

### 3.3. Antimicrobial Resistance

**Figure 1:**
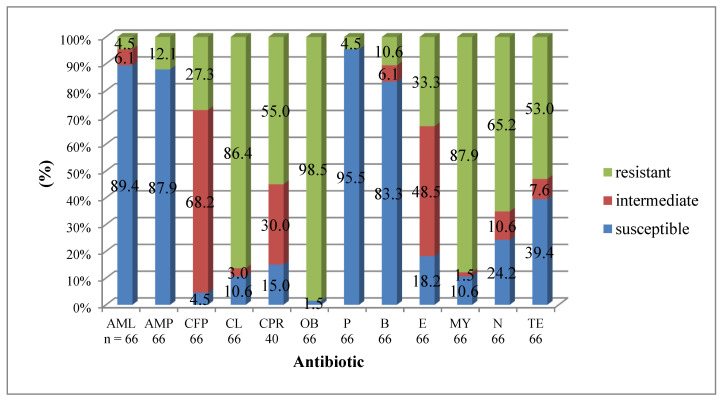
Antimicrobial susceptibility results for enterococci: AML—amoxicillin (25 µg), AMP—ampicillin (10 µg), CFP—cefoperazone (30 µg), CL—cephalexin (30 µg), CPR—cefapirin (30 µg), OB—cloxacillin (5 µg), P—penicillin G (10 u), B—bacitracin (10 u), E—erythromycin (15 µg), MY—lincomycin (15 µg), N—neomycin (30 µg), TE—tetracycline (30 µg).

The results of the tests on the susceptibility of enterococcal isolates to antibiotics are presented in Figure 1. The isolates were predominantly resistant to cloxacillin (98.5%), lincomycin (87.9%), cefalexin (86.4%), neomycin (65.2%), cefapirin (55%) and tetracycline (53%). Most *Enterococcus* isolates were susceptible to penicillin (95.5%), amoxicillin (89.4%), ampicillin (87.9%), and bacitracin (83.3%). Almost 70% of enterococci showed intermediate susceptibility to cefoperazone, approximately 50% to erythromycin, and 30% to cefapirin.

Table 8 shows the prevalence of resistance profiles among enterococci.

Of the 80 *Enterococcus* spp. isolates obtained from the mammary glands of cows, 49 (61.3%) were found to carry at least one of the tested tetracycline, erythromycin and vancomycin resistance genes (Table 9). The most prevalent gene was *tet*(M) (n = 41 isolates), followed by *erm*(B) (n = 24), *tet*(L) (n = 20), *tet*(O) (n = 6), *vanC2/3* (n = 2) and *vanC1* (n = 1). The majority of isolates carrying the *erm*(B) gene (87.5%) belonged to the *E. faecalis* species (Table 10, Table 11 and Table 12). The *tet*(K), *erm*(A), *erm*(C), *mef*(A), *vanA* and *vanB* genes were not detected in the tested isolates. Twenty-eight enterococcal isolates (35%) harboured two or more resistance genes (Table 9).

Most *Enterococcus* isolates carrying the macrolide antibiotic resistance gene *erm*(B) (95.83%; 23/24) were found to have at least one tetracycline resistance gene (Table 9).

The number of isolates exhibiting phenotypic resistance did not exactly correspond to the number of isolates harbouring the selected resistance genes (Table 13). The *erm*(B) gene was detected in 90.9% (20/22) of enterococcal isolates that were phenotypically resistant to erythromycin, as well as in the only isolate that showed intermediate resistance to this antibiotic (Table 13). The other macrolide resistance genes tested (*erm*(A), *erm*(C) and *mef*(A)) were not detected. The *tet*(M) gene was present in 94.3% (33/35) of enterococcal isolates that were phenotypically resistant to tetracycline. In most of these isolates, the *tet*(M) gene was present either alone (45.7%) or in combination with the *tet*(L) gene (40.0%) (Table 13).

The MIC values for vancomycin were 4 µg/mL for one *E. gallinarum* isolate carrying the *vanC1* gene and two *E. casseliflavus* isolates carrying the *vanC2/3* gene (vancomycin-susceptible isolates according to the CLSI guidelines) [43].

## 4. Discussion

### 4.1. The Incidence of Enterococcal Species Causing Mastitis in Cows

In recent years, interest in enterococci has grown. On the one hand, this is due to the widespread involvement of these opportunistic pathogens in causing infections in humans and animals and their increasing resistance to antibiotics used in human and veterinary medicine. On the other hand, it is due to the development of microbial identification techniques. Our previous study showed that enterococci were isolated from 2.8% of clinical and subclinical cases of mastitis in Polish herds [5]. Of the eighty isolates examined in the current study, 78.8% belonged to the two main species of enterococci: *E. faecalis* (65%) and *E. faecium* (13.8%). These two species were isolated at a similar frequency from the milk of cows with mastitis in South Korea (86.4% vs. 13.6%) [46]. In this study, 21.2% of the isolates belonged to other species of enterococci. Enterococcal species such as *E. durans*, *E. gallinarum*, *E. casseliflavus*, *E. avium* and *E. hirae* have occasionally been isolated from bovine milk and human infections [11,47]. Generally, *E. faecalis* is more prevalent in infections than *E. faecium*, which is due to its significantly higher prevalence in the environment and greater number of virulence factors [48]. Recently, an increase in *E. faecium*-related infections has been observed in human medicine, potentially due to its ability to acquire new resistance or virulence factors [49,50].

### 4.2. Virulence Factor Genes

The *E. faecalis* isolates examined in the study harboured multiple virulence genes (from two to six), whereas the only virulence gene detected in the *E. faecium* isolates was the *efaA*_fm_ gene. Previous studies have revealed that some virulence genes (*ace*, *asa1*, *esp* and *gelE*) can occur in *E. faecium* isolates; however, *E. faecalis* isolates harboured significantly more virulence factors than *E. faecium* [46,51,52]. Interestingly, research with *Enterococcus* spp. isolated from artisanal dairy products has reported that non-*faecalis*/non-*faecium* enterococcal isolates carried at least three virulence genes, similar to *E. faecalis* and *E. faecium* [53]. In the present study, the absence of tested virulence genes in non-*faecalis* isolates may be due to the relatively small number of isolates compared to *E. faecalis* isolates.

The EfaA protein is an adhesin found in the serum of patients with endocarditis [54], which may facilitate the adhesion of bacterial cells to myocardial cells [55]. The presence of genes encoding the EfaA protein has been confirmed in various enterococcal species, including *E. faecalis*, *E. faecium*, *E. durans* and *E. solitarius* [54]. All *E. faecalis* and *E. faecium* isolates examined in our study were found to carry the *efaA*_fs_ and *efaA*_fm_ genes, respectively. These genes were also detected in all *E. faecalis* and *E. faecium* isolates from human infections, milk and dairy products [51,56] and in 94.6% of *E. faecalis* isolates from cows with mastitis [57]. These results may indicate that enterococci, which cause mastitis in cows, have the potential to cause disease in humans as well.

Our studies confirmed the observations of other authors regarding the frequent presence of the *ace* gene, which encodes the Ace adhesion protein, and the *gelE* gene [52,56,58,59], which is involved in gelatinase and biofilm production in *E. faecalis* [18]. In our studies, the *ace* (98.1%) and *gelE* (86.5%) genes, alongside the *efaA* gene, were the genes most frequently found in *E. faecalis* isolates from clinical and subclinical bovine mastitis, similarly to previous studies conducted on Turkish isolates [57].

In our study, neither the *gelE* nor the *ace* gene was detected in *E. faecium* isolates. However, some authors have found one *gelE*-positive *E. faecium* isolate in bovine mastitic milk (1/11; 9.1%) [46]. Other authors have reported that the gene was detected more frequently in *E. faecalis* than in *E. faecium* [59]. The *E. faecalis* Ace protein has been shown to be equivalent to the Acm protein (*acm* gene) in *E. faecium*, and an additional *E. faecium* Scm protein (*scm* gene) has been identified [55]. However, some authors have reported detecting the *ace* gene in two *E. faecium* isolates (2/50; 4%) from the milk of cows with mastitis [60].

The aggregating substance found in enterococci is an adhesin encoded by genes located on pheromone-dependent plasmids, including the *asa1* gene [16]. In our study, the *asa1* gene was present in most of the *E. faecalis* isolates tested (63.5%), a result consistent with those obtained from raw milk samples (67.1–100%) [46,56,61] and from the milk of cows with mastitis (71.4%) [46]. However, in contrast to our own studies, the *asa1* gene was also detected in *E. faecium* isolates and other species (e.g., *E. gallinarum* and *E. hirae*) from bovine milk, including mastitic milk [20,46,60].

In addition to its role in adhesion, Esp is also believed to play a role in evading the host’s immune response, which is an important factor in disease development [62]. Although the *esp* gene is not essential for biofilm formation [17,18], *esp*-positive clinical *E. faecalis* isolates produce a greater quantity of biofilm than isolates lacking this gene [18]. In the present study, the *esp* gene was detected in 57.7% of *E. faecalis* isolates from the mammary glands of cows with mastitis. These results are comparable to those of previous studies [46,57,60]. In contrast, the gene was present in 4% of *E. faecium* isolates from clinical mastitis cases in China [60].

Cytolysin is a pore-forming exotoxin that affects (lyses) both eukaryotic and prokaryotic cells. Produced by haemolytic strains of *E. faecalis*, it is associated with high virulence and a higher death rate in animal and human infections [63]. Cytolysin production by enterococci (mainly *E. faecalis*) is a multistep process involving an operon composed of eight genes [16,64]. One of these genes is *cylA*, which is responsible for expressing protein A, an enzyme activator [15,16]. Some previous studies have demonstrated the presence of the *cylA* gene in 1.8% and 30% of *E. faecalis* isolates from clinical and subclinical cases of bovine mastitis [46,57]. In this study, the *cylA* gene was present in 17.3% of *E. faecalis* isolates and none of the *cylA*-positive isolates caused complete lysis of red blood cells (β-haemolysis). Other authors have also observed a discrepancy between phenotypic and genotypic expression of cytolysin [46,56,58]. This may be due to the fact that the genes encoding cytolysin are selectively expressed [65]. Furthermore, in the current study, we used agar supplemented with 5% sheep blood for the examination of haemolysin. However, certain strains of *E. faecalis* can produce haemolysin/cytolysin, which acts on human, rabbit and horse erythrocytes but not sheep ones [66]. This is associated with different levels of susceptibility of erythrocytes from various species to hemolysin-mediated lysis [65]. For these reasons, to demonstrate the full ability of bovine enterococci to lyse red blood cells, it is justified to determine haemolysis using an agar containing 5% horse blood.

The *hyl*_fm_ gene, which is associated with the production of hyaluronidase, is considered a potential virulence factor in clinical isolates of *E. faecium*. It is more prevalent in vancomycin-resistant strains than in vancomycin-susceptible strains [67], likely due to its location on large plasmids that may carry glycopeptide resistance genes [68]. In this study, the *hyl*_fm_ gene was not detected in the enterococci, consistent with previous studies of isolates from cow’s milk [20,52,60] and food [69].

### 4.3. Antimicrobial Resistance of Enterococci Isolated from Bovine Mastitis

The steady increase in bacterial drug resistance observed over the past two decades has prompted various organisations, including the WHO and the European Commission, to take action to reduce and monitor antibiotic use. While most EU countries have experienced a decline in antimicrobial sales, Poland ranks second in terms of total sales of preparations used for food-producing animals and average consumption of antimicrobial substances for these animals (mg/Population Correction Unit; mg/PCU) [70,71]. In recent years, the highest sales were for penicillins, tetracyclines and macrolides [71], which are antibiotics that are often used in preparations to treat bovine mastitis caused by *Streptococcus* species [23,72]. The resistance to macrolides, lincosamides and tetracyclines observed in our study may be due to the intensive and long-term use of antibiotics for treating mastitis during the lactation and dry periods, which has contributed to the selection of resistant strains [26].

The strains were tested for their susceptibility to the antibiotics commonly used to treat mastitis in cows, which is caused by Gram-positive, catalase-negative cocci. As expected, vast majority of *Enterococcus* spp. isolates were resistant to certain β-lactam antibiotics, such as cloxacillin and cephalexin. A low percentage of the isolates were resistant to penicillin G, amoxicillin and ampicillin. Previous studies conducted in Poland, Finland, the USA, Brazil and China also reported low percentages of resistance to penicillin (0–5.05%) and ampicillin (0%) [4,20,60,73,74,75]. Other studies have reported higher percentages of penicillin-resistant enterococci, at 62.3% in China [76] and 64.8% in Korea [77] compared with those isolated from Polish dairy cows. In our own studies, a significant proportion of enterococcal isolates were found to be resistant to lincomycin (87.9%), which is consistent with the results of previous research [4,76], and to neomycin (65.2%). However, in a Korean study, the percentage of isolates resistant to neomycin was lower (1.2%) [46]. The very high resistance rates to cloxacillin, cephalexin and lincomycin are due to the intrinsic resistance of enterococci to these antibiotics. *Enterococcus* spp. exhibit intrinsic resistance to low concentrations of β-lactams (mainly cephalosporins), aminoglycosides and clindamycin (lincosamide), as well as acquired resistance to high concentrations of clindamycin and aminoglycosides [32].

In our study, over half of the enterococcal isolates (53%) were resistant to tetracycline, indicating a lower resistance rate than that reported by other authors in Poland (82%) [4]. Even lower percentages of tetracycline-resistant isolates were reported in the USA (22.5%) and Brazil (27.3%) [20,73].

Many genes conferring resistance to tetracyclines have been identified. In Gram-positive cocci, the most common are *tet*(M) and *tet*(O), which encode proteins that protect ribosomes from the action of tetracycline, and *tet*(K) and *tet*(L), which encode proteins that pump the antibiotic out of the bacterial cell [78]. Almost all tetracycline-resistant isolates carried the *tet*(M) gene (33/35; 94.3%). The second most frequently detected gene in tetracycline-resistant isolates was the *tet*(L) gene (45.7%). The *tet*(O) gene was detected in 8.6% of isolates. Similar results were obtained in studies of high-level erythromycin-resistant (HLER) *E. faecalis* isolated from bulk tank milk of dairy companies in Korea [61]. In our own studies, no tetracycline resistance associated with the presence of the *tet*(K) gene was found. Other authors reported that this gene was rare in enterococci [79]. However, a surprisingly high frequency of the *tet*(K) gene was reported in other studies, where the *tet*(K) gene was found in 97.2% and 25% of tetracycline-resistant *E. faecalis* isolates from subclinical and clinical mastitis cases, respectively [80,81]. The *tet*(K) gene is generally believed to be primarily found in Gram-positive bacteria belonging to the *Staphylococcus* genus [82]. The presence of this gene in enterococci could suggest that they have the ability to acquire resistance genes from other species of bacteria.

In our own studies, the prevalence of erythromycin resistance among enterococci was 33.3%, while the percentage of susceptible isolates was 18.2%. Studies conducted in Finland found that 19% of enterococcal isolates were resistant to this antibiotic [74]. In Korea, 57.1% of isolates were resistant and 22.86% were susceptible [77]. In China, resistance to erythromycin was very high, at 83.8% of isolates [60].

There are two mechanisms of erythromycin resistance in bacteria. The first involves ribosome modification by a methylase encoded by *erm* genes. The second mechanism involves a drug efflux pump, which is a hydrophobic membrane-bound protein encoded by the *mef* gene [83]. In this study, *erm*(B) was the only gene conferring erythromycin resistance among the isolates, with 90.9% of the erythromycin-resistant isolates carrying this gene. Two isolates did not contain any of the *erm* or *mef*(A) genes tested. Similar results were obtained by Jensen et al. [84], who demonstrated the presence of the *erm*(B) gene in most erythromycin-resistant enterococcal isolates from cattle, but not the *erm*(A) or *erm*(C) genes. In China, the majority of enterococcal isolates from clinical mastitis carried the *erm*(B) gene (95.5%), although *erm*(A)-positive (1%) and *erm*(C)-positive (7.1%) isolates were also detected [60]. Another study found that the *erm*(A) gene was not present in erythromycin-resistant *E. faecalis* isolates from the milk of cows with subclinical mastitis, which is consistent with our own findings [80].

Previous studies have shown that enterococcal isolates from cows with mastitis often exhibit greater acquired resistance to tetracycline and erythromycin than to other tested antimicrobials [46,60,80]. These resistance genes are frequently found on the same mobile genetic element, suggesting that tetracycline-resistant strains may play a role in the dissemination of erythromycin resistance [85]. In this study, almost all *erm*(B)-positive isolates carried at least one *tet* gene.

Our own study, as well as those of other authors [60,81,82], has revealed an inconsistency between the presence of resistance determinants and phenotypic antibiotic resistance. A variety of mechanisms cause antibiotic resistance, including the presence of specific genes or changes in bacterial metabolism [81]. In this study, several isolates were phenotypically resistant to erythromycin or tetracycline, but genotypically susceptible. This suggests that these isolates may carry antibiotic resistance genes that were not tested for. An example of such a gene is the *tetS* gene, which confers resistance to tetracycline [60,81]. In this study, two isolates that were phenotypically susceptible carried tetracycline-resistance genes. Previous studies, including those involving WGS, have also detected *tet* or *erm* genes in isolates that are phenotypically susceptible; however, the authors demonstrated that it was caused by incorrect phenotypic antibiotic testing results or the presence of silenced antimicrobial resistance genes [86,87]. The absence of antimicrobial resistance gene expression may be due to mutation, a non-functional promoter, integrons or negative transcriptional regulators [88], which were not examined in this study. Another reason for the discrepancy between the presence of resistance genes and phenotypic resistance to antibiotics may be high detection limits (μg/mL). The detection limits determined for most antibiotics used in the treatment of mastitis in cows showed higher values for the disc diffusion method compared to other antibiotic detection methods, such as Delvotest SP and Penzym S 100 (e.g., oxacillin, ampicillin, cefotaxime, cefoperazone, cefquinome and ceftazidime) or Delvotest SP (e.g., chlortetracycline, erythromycin and bacitracin) [89].

Administering sub-therapeutic doses of antibiotics to farm animals through their feed has contributed to the development and spread of multidrug resistance. The glycopeptide antibiotic avoparcin was used in dairy cows in European Union countries until 1997. This resulted in the emergence of *Enterococcus* spp. strains that were resistant to vancomycin, an important drug used to treat hospital-acquired infections caused by enterococci and a last-resort antibiotic for treating infections caused by methicillin-resistant *S. aureus* [90]. Ten gene clusters conferring vancomycin resistance have been identified in enterococci. The VanA and VanB clusters are the most prevalent among those isolated from hospital-acquired infections, particularly in *E. faecium*. These clusters are typically transferred via mobile genetic elements that can integrate into the chromosomes or plasmids [50] of vancomycin-susceptible enterococci, other pathogenic bacteria (e.g., *Staphylococcus aureus*) or non-pathogenic microorganisms that inhabit the gastrointestinal tract of humans and animals [91]. In our study, the genes responsible for acquired resistance to high vancomycin concentrations (*vanA* and *vanB*) were not detected. However, the absence of these genes in the examined isolates does not exclude their presence in other Polish herds or at different times. An *E. faecalis* isolate carrying the *vanB* gene was found in cow’s milk in Bangladesh [81]. In our studies, three isolates exhibited the VanC phenotype, characterised by constitutive or induced resistance to low concentrations of vancomycin (MIC = 4–16 µg/mL). This phenotype occurs naturally in most *E. gallinarum* (*vanC1 gene*) and *E. casseliflavus* (*vanC2/3*) isolates [35,44]. These *vanC*-positive species remain epidemiologically relevant reservoirs of glycopeptide tolerance.

This study has some limitations. Although efforts were made to avoid duplicating isolates from the same herds, it should be noted that the number of non-*faecalis* isolates examined in the study was fairly limited. Therefore, the small sample size limits the statistical power available for robust comparisons between species and detailed analysis of less common enterococcal species. The same situation also applies to the relatively small number of *Enterococcus* spp. isolates from subclinical mastitis cases (n = 7) compared to the number of clinical cases (n = 45). Therefore, conclusions regarding species comparisons and mastitis type associations are exploratory rather than definitive. Furthermore, the identification of the isolates was based on biochemical and serological methods, as well as standard PCR techniques. The use of more advanced methods based on 16S rDNA sequencing, which remains the gold standard in microbial identification, or the MALDI-TOF MS technique, would also enable the identification of species other than *E. faecalis* and *E. faecium*. A further limitation of the study is that no investigation was made of the formation of biofilms. The ability of *Enterococcus* spp. to form biofilms is an important virulence factor. At least two biofilm formation-associated virulence genes (*esp* and *gelE*) were found in the majority of *E. faecalis* isolates tested. Therefore, it would be worthwhile conducting such a study in the future. Phenotypic confirmation of the ability to form biofilms would significantly strengthen the virulence assessment.

## 5. Conclusions

This study describes the virulence and antibiotic resistance gene profiles of enterococci isolated from clinical and subclinical cases of bovine mastitis in Poland. Our study revealed that *E. faecalis* showed a higher virulence gene burden. We were unable to find a correlation between the presence of virulence genes and the incidence of subclinical or clinical mastitis in cows. Antibiotic-resistant isolates of enterococci were found in the milk of cows with mastitis in Poland. These isolates may serve as a potential reservoir of resistance genes (*erm*, *tet*) for other bacteria colonising the human gastrointestinal tract. However, this theoretical risk is supported by molecular evidence rather than demonstrated transmission. None of the *Enterococcus* spp. examined in this study exhibited the *vanA* or *vanB* genotypes that correspond to acquired resistance to high concentrations of vancomycin. Nevertheless, the absence of these genes in the tested isolates does not exclude their presence in other Polish herds or at different times. Due to the resistance of enterococci, the decision on treatment should be based on the results of a microbiological examination and an antibiogram. Enterococci exhibit natural resistance to bacitracin, cloxacillin, cephalosporins and lincomycin. Antibiotics from the penicillin group (e.g., penicillin, amoxicillin, ampicillin) are often effective, unless confirmed by an antibiogram. Therefore, it is important to accurately identify Gram-positive, catalase-negative cocci isolated from the mammary glands of cows and to monitor their antibiotic susceptibility in different countries, including Poland. Comprehensive monitoring of antimicrobials used in dairy production is required to address the resistance of enterococci to antibiotics.

## Figures and Tables

**Table 1:** Reference strains and isolates from the *Enterococcus* and *Staphylococcus* genera used to detect virulence and antimicrobial resistance genes.

Strain/Isolate	Virulence Genes	Antibiotic Resistance Determinants
*E. faecalis* ATCC 29212	*ace*, *asa1*, *cylA*, *gelE*, *efaA*_fs_	*tet*(L), *tet*(M), *tet*(O)
*E. faecalis* ATCC 51299	*ace*, *asa1*, *cylA*, *esp*, *gelE*, *efaA*_fs_	*erm*(B), *vanB*
*E. faecium* ATCC 19434	*gelE*, *efaA*_fm_	nd
*E. faecium* ATCC 51559	*esp*, *hyl*, *efaA*_fm_	*vanA*
*E. gallinarum* ATCC 49573	nd	*vanC1*
*E. casseliflavus* ATCC 25788	nd	*vanC2/3*
*S. aureus* 192	-	*erm*(C)
*S. aureus* 368	-	*tet*(K)

Notes: nd—not detected.

**Table 2:** Sequences of primers and cycling conditions used to identify enterococci isolated from the milk of dairy cows with mastitis by PCR.

Genus/Species	Gene	Description	Oligonucleotide Name and Sequence (5′→3′)	Size of PCR Product (bp)	MgCl_2_ Concentration (mM)	Reference
*Enterococcus*	*tuf*	Elongation factor EF-Tu	Ent1: TACTGACAAACCATTCATGATG	112 ^a^	5	[35]
			Ent2: AACTTCGTCACCAACGCGAAC			
*E. faecalis*	*ddl_E.faecalis_*	D-Ala:D-Ala ligase	E_1_: ATCAAGTACAGTTAGTCT	941 ^b^	2	[36]
			E_2_: ACGATTCAAAGCTAACTG			
*E. faecium*	*ddl_E.faecium_*	D-Ala:D-Ala ligase	Ecium-F: CGCAGAGCATGAAGTGTCCA	557 ^c^	4	[37]
			Ecium-R2: CTTCTCGGTTTTCTGCTTTTGTA			
*E. gallinarum*	*vanC1*	VanC phenotype	C_1_: GGTATCAAGGAAACCTC	822 ^b^	1.5	[36]
			C_2_: CTTCCGCCATCATAGCT			
*E. casseliflavus* ^d^	*vanC2/3*	VanC phenotype	D_1_: CTCCTACGATTCTCTTG	439 ^b^	1.5	[36]
			D_2_: CGAGCAAGACCTTTAAG			

Notes: ^a^ Cycling conditions: 95 °C (180 s); 35 cycles of 95 °C (30 s), 55 °C (30 s), 72 °C (60 s); final extension 72 °C (420 s). ^b^ Cycling conditions: 94 °C (120 s); 30 cycles of 94 °C (60 s), 54 °C (60 s), 72 °C (60 s); final extension 72 °C (600 s). ^c^ Cycling conditions: 95 °C (300 s); 30 cycles of 95 °C (30 s), 65 °C (30 s), 72 °C (30 s); additional annealing 65 °C (30 s); final extension 72 °C (300 s). ^d^ *E. casseliflavus* and *E. flavescens* were found to be the same species, with the recommendation that the name *E. casseliflavus* be retained [38].

**Table 3:** Sequences of PCR primers and cycling conditions used to amplify fragments of virulence factor genes of *Enterococcus* spp. isolated from bovine mastitic milk.

Gene	Description	Oligonucleotide Name and Sequence (5′→3′)	Size of PCR Product (bp)	MgCl_2_ Concentration (mM)	Reference
*ace*	collagen-binding protein Ace	Ace 1: GGAATGACCGAGAACGATGGC	616 ^a^	1.5	[39]
		Ace 2: GCTTGATGTTGGCCTGCTTCCG			
*asa1*	aggregation substance AS	Asa 11: CACGCTATTACGAACTATGA	375 ^b^	1.5	[15,39]
		Asa 12: TAAGAAAGAACATCACCACGA			
*cylA*	cytolysin Cyl	Cyt I: ACTCGGGGATTGATAGGC	688 ^b^	3	[15,39]
		Cyt IIb: GCTGCTAAAGCTGCGCTT			
*efaA*_fs_	*Enterococcus faecalis* endocarditis antigen	EfaAF: AGGCGGAAATGGCTGGTTTA	569 ^c^	2	this study
		EfaAR: TGGCAAGGGAGTCTGTGAAA			
*efaA*_fm_	*Enterococcus faecium* endocarditis antigen	EfaA1: CGCCTGACCAAATGAAAGCA	361 ^c^	2	this study
		EfaA2: AGCCACACCTTCCTTCCTTAC			
*esp*	enterococcal surface protein Esp	Esp 14F: AGATTTCATCTTTGATTCTTGG	510 ^b^	3	[15,39]
		Esp 12R: AATTGATTCTTTAGCATCTGG			
*gelE*	gelatinase GelE	Gel 11: TATGACAATGCTTTTTGGGAT	213 ^b^	5	[15,39]
		Gel 12: AGATGCACCCGAAATAATATA			
*hyl*_fm_	hyaluronidase Hyl	Hyl n1: ACAGAAGAGCTGCAGGAAATG	276 ^b^	2.5	[15,39]
		Hyl n2: GACTGACGTCCAAGTTTCCAA			

Notes: ^a^ Cycling conditions: 95 °C (600 s); 30 cycles of 94 °C (30 s), 58 °C (30 s), 72 °C (30 s); final extension 72 °C (600 s). ^b^ Cycling conditions: 95 °C (600 s); 30 cycles of 94 °C (30 s), 56 °C (30 s), 72 °C (30 s); final extension 72 °C (600 s). ^c^ Cycling conditions: 95 °C (600 s); 30 cycles of 94 °C (60 s), 52 °C (60 s), 72 °C (60 s); final extension 72 °C (600 s).

**Table 4:** Sequences of the PCR primers and the cycling conditions used to amplify fragments of the antimicrobial resistance genes in the enterococci that were isolated from the milk of cows with mastitis.

Gene	Antimicrobial Resistance	Oligonucleotide Name and Sequence (5′→3′)	Size of PCR Product (bp)	MgCl_2_ Concentration (mM)	Reference
*erm*(A)	macrolides	ermAF: TCTAAAAAGCATGTAAAAGAA	645 ^a^	3	[40,41]
		ermAR: CTTCGATAGTTTATTAATATTAGT			
*erm*(B)	macrolides	ermBF: GAAAAGGTACTCAACCAAATA	639 ^a^	2	[24,40]
		ermBR: AGTAACGGTACTTAAATTGTTTAC			
*erm*(C)	macrolides	ermCF: TCAAAACATAATATAGATAAA	642 ^b^	3	[40,41]
		ermCR: GCTAATATTGTTTAAATCGTCAAT			
*mef*(A)	macrolides	Fw: AGTATCATTAATCACTAGTGC	348 ^a^	3	[24,40]
		Rv: TTCTTCTGGTACTAAAAGTGG			
*tet*(K)	tetracyclines	tetK 1: GTAGCGACAATAGGTAATAGT	360 ^c^	1.5	[42]
		tetK 2: GTAGTGACAATAAACCTCCTA			
*tet*(L)	tetracyclines	tetLF: TGAACGTCTCATTACCTG	993 ^d^	2.5	[41]
		tetLR: ACGAAAGCCCACCTAAAA			
*tet*(M)	tetracyclines	tetMF: TTATCAACGGTTTATCAGG	397 ^b^	1.5	[41]
		tetMR: CGTATATATGCAAGACG			
*tet*(O)	tetracyclines	tetOF: AACTTAGGCATTCTGGCTCAC	515 ^e^	1.5	[41]
		tetOR: TCCCACTGTTCCATATCGTCA			
*vanA*	glycopeptides	A_1_: GGGAAAACGACAATTGC	732 ^f^	1.5	[36]
		A_2_: GTACAATGCGGCCGTTA			
*vanB*	glycopeptides	B_1_: ATGGGAAGCCGATAGTC	635 ^f^	1.5	[36]
		B_2_: GATTTCGTTCCTCGACC			
*vanC1*	glycopeptides	C_1_: GGTATCAAGGAAACCTC	822 ^f^	1.5	[36]
		C_2_: CTTCCGCCATCATAGCT			
*vanC2/3*	glycopeptides	D_1_: CTCCTACGATTCTCTTG	439 ^f^	1.5	[36]
		D_2_: CGAGCAAGACCTTTAAG			

Notes: ^a^ Cycling conditions: 93 °C (180 s); 35 cycles of 93 °C (60 s), 52 °C (60 s), 72 °C (60 s); final extension 72 °C (300 s). ^b^ Cycling conditions: 94 °C (600 s); 35 cycles of 94 °C (60 s), 46 °C (60 s), 72 °C (60 s); final extension 72 °C (300 s). ^c^ Cycling conditions: 94 °C (180 s); 30 cycles of 94 °C (30 s), 55 °C (30 s), 72 °C (30 s); final extension 72 °C (240 s). ^d^ Cycling conditions: 94 °C (600 s); 35 cycles of 94 °C (60 s), 50 °C (60 s), 72 °C (60 s); final extension 72 °C (300 s). ^e^ Cycling conditions: 94 °C (600 s); 35 cycles of 94 °C (60 s), 55 °C (60 s), 72 °C (60 s); final extension 72 °C (300 s). ^f^ Cycling conditions: 94 °C (120 s); 30 cycles of 94 °C (60 s), 54 °C (60 s), 72 °C (60 s); final extension 72 °C (600 s).

**Table 5:** Types of haemolysis produced by the 80 enterococcal isolates obtained from cows with mastitis.

Species/Genus	Haemolysis No. of Isolates (%)		
	α	β	γ
*E. faecalis*	7/52 (13.5)	0/52 (0)	45/52 (86.5)
*E. faecium*	11/11 (100)	0/11 (0)	0/11 (0)
*Enterococcus* spp.	13/17 (76.5)	0/17 (0)	4/17 (23.5)
Total	31/80 (38.8)	0/80 (0)	49/80 (61.3)

**Table 6:** Occurrence of virulence determinants in bovine *E. faecalis* isolates (n = 52) from clinical and subclinical mastitis.

Virulence Gene	Clinical Mastitis (n = 45), n (%)	Subclinical Mastitis (n = 7), n (%)	*p* *	Total (n = 52), n (%)
*ace*	44 (97.8)	7 (100)	0.969	51 (98.1)
*asa1*	27 (60.0)	6 (85.7)	0.556	33 (63.5)
*cylA*	7 (15.6)	2 (28.6)	0.494	9 (17.3)
*efaA*_fs_	45 (100)	7 (100)	0.999	52 (100)
*esp*	25 (55.6)	5 (71.4)	0.693	30 (57.7)
*gelE*	38 (84.4)	7 (100)	0.770	45 (86.5)
*hyl*	0 (0)	0 (0)	0.154	0 (0)

* *p* ≤ 0.05 was considered statistically significant.

**Table 7:** Genotypes of *E. faecalis* isolated from the milk of dairy cows with mastitis (52 isolates).

Profile	Genes	Isolates	
		Number	%
1	*ace asa1 cylA efaA*_fs_ *esp gelE*	8	15.4
2	*ace asa1 efaA*_fs_ *esp gelE*	11	21.2
3	*ace asa1 cylA efaA*_fs_	1	1.9
4	*ace asa1 efaA*_fs_ *gelE*	9	17.3
5	*ace efaA*_fs_ *esp gelE*	9	17.3
6	*ace asa1 efaA*_fs_	4	7.7
7	*ace efaA*_fs_ *esp*	2	3.8
8	*ace efaA*_fs_ *gelE*	7	13.5
9	*efaA*_fs_ *gelE*	1	1.9

**Table 8:** Antimicrobial resistance (AMR) profiles among *Enterococcus* spp. isolates (n = 66).

Antibiotype	*E. faecalis* n (%)	*E. faecium* n (%)	Other *Enterococcus* spp. n (%)
N*-β-lactams*-MY*-E-B-TE	3 (6.5)	1 (11.1)	nd *
N*-β-lactams*-MY*-E-B	1 (2.2)	nd	nd
N*-β-lactams*-MY*-E-TE	14 (30.4)	nd	nd
N*-β-lactams*-MY*-TE	13 (28.3)	nd	nd
β-lactams*-MY*-E-TE	1 (2.2)	1 (11.1)	nd
N*-β-lactams*-MY*	9 (19.6)	nd	nd
N*-β-lactams*-E	nd	1 (11.1)	nd
β-lactams*-MY*-TE	1 (2.2)	nd	1 (9.1)
N*-β-lactams*	nd	1 (11.1)	nd
β-lactams*-MY*	4 (8.7)	1 (11.1)	7 (63.6) (including *E. gallinarum* and *E. casseliflavus*)
β-lactams*	nd	4 (44.4)	2 (18.2)
MY*	nd	nd	1 (9.1)

* nd—not detected. N, neomycin (aminoglycoside); β-lactams (amoxicillin, ampicillin, cefoperazone, cephalexin, cefapirin, cloxacillin, penicillin G); My, lincomycin (lincosamide); E, erythromycin (macrolide); B, bacitracin (polypeptide); TE, tetracycline. * *Enterococcus* spp. are intrinsically resistant to cephalosporins and exhibit low-level resistance to β-lactams, aminoglycosides and lincosamides [32,45].

**Table 9:** Occurrence of resistance genes and their combinations in all enterococci examined in the study (n = 80).

Profile	Genes	Isolates	
		Number	%
1	*tet*(M)	15	18.8
2	*tet*(O)	2	2.5
3	*tet*(L) *tet*(M)	3	3.8
4	*tet*(L) *tet*(O)	1	1.3
5	*tet*(M) *tet*(O)	1	1.3
6	*erm*(B)	1	1.3
7	*tet(L) erm(B)*	1	1.3
8	*tet(M) erm(B)*	7	8.8
9	*tet*(L) *tet*(M) *erm*(B)	13	16.3
10	*tet*(L) *tet*(M) *tet*(O) *erm*(B)	2	2.5
11	*vanC1*	1	1.3
12	*vanC2/3*	2	2.5
13	-	31	38.8
Total		80	100

**Table 10:** Occurrence of resistance genes and their combinations in *E. faecalis* isolates (n = 52).

Profile	Genes	Isolates	
		Number	%
1	*tet*(M)	15	28.8
2	*tet*(L) *tet*(M)	2	3.8
3	*tet*(L) *tet*(O)	1	1.9
4	*tet*(M) *tet*(O)	1	1.9
5	*tet*(L) *tet*(M) *tet*(O) *erm*(B)	2	3.8
6	*tet*(L) *tet*(M) *erm*(B)	12	23.1
7	*tet*(L) *erm*(B)	1	1.9
8	*tet*(M) *erm*(B)	6	11.5
9	-	12	23.1

**Table 11:** Occurrence of resistance genes and their combinations in *E. faecium* isolates (n = 11).

Profile	Genes	Isolates	
		Number	%
1	*tet*(L) *tet*(M)	1	9.1
2	*tet*(L) *tet*(M) *erm*(B)	1	9.1
3	*erm*(B)	1	9.1
4	-	8	72.7

**Table 12:** Occurrence of resistance genes and their combinations in *Enterococcus* spp. isolates other than *E. faecalis* and *E. faecium* (n = 17).

Profile	Genes	Isolates	
		Number	%
1	*tet*(O)	2	11.8
2	*tet*(M) *erm*(B)	1	5.9
3	*vanC1* (*E. gallinarum*)	1	5.9
4	*vanC2* (*E. casseliflavus*)	2	11.8
5	-	11	64.7

**Table 13:** Prevalence of erythromycin and tetracycline resistance phenotypes and genotypes among *Enterococcus* spp. isolates (n = 80).

Antibiotic/Gene	Antibiotic Susceptibility Category				Total
	Susceptible n (%)	Intermediate n (%)	Resistance n (%)	Undetermined * n (%)	
erythromycin	12	32	22	14	80
*erm*(A)	0 (0.0)	0 (0.0)	0 (0.0)	0 (0.0)	0 (0.0)
*erm*(B)	0 (0.0)	1 (3.1)	20 (90.9)	3 (21.4)	24 (30.0)
*erm*(C)	0 (0.0)	0 (0.0)	0 (0.0)	0 (0.0)	0 (0.0)
*mef*(A)	0 (0.0)	0 (0.0)	0 (0.0)	0 (0.0)	0 (0.0)
tetracycline	26	5	35	14	80
*tet*(K)	0 (0.0)	0 (0.0)	0 (0.0)	0 (0.0)	0 (0.0)
*tet*(L)	0 (0.0)	1 (20.0)	0 (0.0)	0 (0.0)	1 (1.3)
*tet*(L) + *tet*(M)	0 (0.0)	0 (0.0)	14 (40.0)	2 (14.3)	16 (20.0)
*tet*(L) + *tet*(M) + *tet*(O)	0 (0.0)	0 (0.0)	2 (5.7)	0 (0.0)	2 (2.5)
*tet*(L) + *tet*(O)	1 (3.8)	0 (0.0)	0 (0.0)	0 (0.0)	1 (1.3)
*tet*(M)	1 (3.8)	1 (20.0)	16 (45.7)	4 (28.6)	22 (27.5)

* a group of isolates for which antibiotic susceptibility has not been determined.

## Data Availability

The datasets presented in the current study are included in the article. Further inquiries can be directed to the corresponding author.

## Abbreviations

The following abbreviations are used in this manuscript:

AML	Amoxicillin
AMP	Ampicillin
AMR	Antimicrobial resistance
B	Bacitracin
CL	Cephalexin
CFP	Cefoperazone
E	Erythromycin
OB	Cloxacillin
MY	Lincomycin
N	Neomycin
P	Penicillin G
TE	Tetracycline
CPR	Cefapirin
